# USA’s exit from the WHO and freeze on USAID funds globally: its perils and possible opportunities

**DOI:** 10.1093/pubmed/fdaf122

**Published:** 2025-11-18

**Authors:** Srikrishna Sulgodu Ramachandra, Premila Webster

**Affiliations:** Department of Community Medicine, KMC Medical College and Hospital, Mahuawa, Farenda Road, Maharajganj District, Uttar Pradesh 273303, India; Somerville College, University of Oxford, Oxford, England

**Keywords:** USA’s WHO exit, USAID fund freeze, impact on global public health

## Abstract

Two Executive Orders signed by USA’s President Trump on 20 January 2025: one, USA’s exit from World Health Organization (WHO) and other, a 90 days’ freeze on USAID’s funds and activities globally with immediate effect, have far reaching impact on global public health and development. Zero fund contribution from USA (currently, largest fund contributor to WHO), means decreased resources which would negatively impact WHO developmental initiatives globally. The impact of USAID fund freeze is yet to be ascertained. DOGE and Trump administration appear to have based their decision on their ‘America First’ to critically assess US aid to global communities, vis-à-vis the requirement and needs to their own people. However, the modus operandi and modalities of executing these orders overnight, without consulting or appraising the stakeholders was not the best way. It erodes the ethos of commitment to Global Health, International Health and Development.

While World Leaders and Global-Public-Health-Community could consider diplomacy and advocacy channels, urging President Trump to reconsider his decisions, some immediate risk-mitigation and damage-control strategies could be put in place. This could include strengthening and expansion of geopolitical realignments, emergence of new leadership, newer models of programme implementation, technology enabled monitoring and evaluation that can reduce costs of programme implementation and management.

## Introduction

The USA has a significant role in Global Health through multilateral engagement, financial contributions, providing policy guidelines and leadership roles in international organizations. Historically, USA has been a major contributor in shaping global health efforts both through direct initiatives like PEPFAR (President’s Emergency Plan for AIDS Relief) and through its contributions to multilateral institutions like World Health Organization (WHO), Global Fund, Pan American Health Organization, and GAVI, The Vaccine Alliance. The CDC (Centers for Disease Control and Prevention) has been a major support in developing and disseminating large scale public health data and surveillance, prevention guidelines, and policy guidelines.

Among the various Executive Orders signed by the US President Mr Donald Trump, two Executive Orders have a far-reaching impact on Public Health globally. These Executive Orders were signed on 20 January 2025, soon after President Trump took charge of the US Federal Government Administration. One of the Executive Order was for the USA to exit from the WHO membership and the other was a 90 days’ freeze on the US Agency for International Development (USAID) funds and activities globally with immediate effect [1–4].

The Department of Government Efficiency (DOGE) and Trump Administration appear to have based their decision on their ‘America First’ motto to critically assess the US aid flow to global communities, vis-à-vis the requirement and needs to their own people. However, the modus operandi and modalities of executing these Orders overnight, without consulting or appraising the primary and secondary stakeholders, was not the best way of undertaking this assessment. It erodes the ethos of commitment to Global Health, International Health and Development. This also exposes the vulnerabilities related to geopolitical dynamics globally [4].

### USA’s withdrawal from WHO membership

In July 2020 also, during his first term as President, Mr Trump had announced that USA will withdraw from WHO. However, since the process takes more than a year and his successor, President Mr. Joe Biden, was not in favour of it, the process did not happen [5]. On 20 January 2025, President Trump signed the Executive Order for withdrawal of USA’s membership from WHO. This has far reaching repercussions. It also means that, the US funding support to WHO would cease with immediate effect. This will negatively impact the WHO’s international health and development efforts across the globe. It is important to note here that, USA is the largest donor to the WHO, contributing $163 million to $816 million each year, over the past decade alone [6].

WHO supports its member countries in terms of funding, technical support, formulating guidelines, monitoring and evaluation, and surveillance, and even logistics for various Public Health initiatives including polio eradication, tuberculosis control, immunization programmes, maternal and child health programmes, non-communicable diseases, etc. [7]

The Global South, including the African Union and the Indian sub-continent receives substantial support from WHO in terms of aid, technical support, training, and logistic support for various key public health initiatives including programmes on immunization, tuberculosis, HIV/AIDS, malaria, maternal and child health initiatives, and initiatives on non-communicable diseases. WHO also plays an important role in disease surveillance initiatives and disaster risk reduction and mitigation initiatives. So, it is obvious that, with decreased resources, there will be an impact on several of these initiatives. These activities may either be diluted or completely stopped.

It is also important to note that, the CDC, USA, will also not be communicating with the WHO [8], resulting in a cessation of the two-way data flow, which would result in the weakening of the Public Health Systems globally. This definitely increases vulnerability towards impending pandemics and public health disasters in the future. In this era of globalization, no single country is immune to diseases, as ‘diseases do not need passports and visas to travel’. This was very evident from the recent COVID-19 pandemic [9]. Hence, the USA is also not immune to the repercussions of these Executive Orders.

## A 90-day freeze on USAID funds and dismantling of USAID

A retrospective impact evaluation of two decades of USAID interventions and forecasting analysis study published in The Lancet dated 30 June 2025 identifies that higher levels of USAID funding, primarily directed towards Low and Middle Income Countries (LMICs) were associated with a 15% reduction in age-standardized all-cause mortality (contributed in preventing 91 million deaths globally, which includes 30 million deaths among children) and a 32% reduction in under-five mortality. Their forecasting models predict that the current fund cuts would result in more than 14 051 750 (14 million) additional all-age deaths, including 4 537 157 (4.5 million) in children younger than age of 5 years, by 2030 [10].

Though the impact of this US Government Order is far reaching and yet to be completely determined, the immediate effects of this Order were:

(i) Cease of funding support to development sector projects across the globe—especially in the African and the Asian context.
(ii) Cease of support to several training and higher education/fellowship programmes that were supported by USAID, across different universities globally.
(iii) Sudden loss of jobs for those involved in the development sector projects supported by USAID. This includes human resource jobs outside the USA and also several US agencies and jobs in headquarters in the USA and offices in several countries (organizations such as FHI 360, Population Services International, etc.)

It is now (as on 10 March 2025) known that 83% of the USAID funded and supported projects globally were terminated following a critical assessment by the DOGE and President Trump’s Administration. It was announced by the US Secretary of State, Mr Marco Rubio, (dated 10 March 2025), that ~5200 of the USAID’s 6200 global programmes were terminated. The remaining approximately, less than one-fifth of the previous aid portfolio will be taken over by the State Department [11].

### Risk mitigation strategies and damage control modalities

‘Necessity is the mother of invention’: considering the example of the COVID-19 pandemic when several countries, identified newer modalities of executing programmes, innovations were fast-tracked, similarly, it is high time that various LMIC governments focus on raising local funds, looking at further strengthening their geopolitical realignments, etc. This situation could be looked at as an opportunity to explore and identify these unexplored or less explored areas. While World Leaders and the Global Public Health Community could consider diplomacy and advocacy channels and urge President Trump to review his decisions, some of the immediate risk mitigation and damage control strategies could include:

### Exploring and further strengthening geo-political realignment

Further strengthening coalition between the BRICS Nations (Brazil, Russia, India, China, South Africa, Egypt, Ethiopia, Iran, United Arab Emirates, and Algeria), strengthening coalition between the South Asian Association for Regional Cooperation Nations, expanding and further impetus on the South–South Cooperation become more important and contextual as part of the geopolitical realignment strategies.

### Emerging new leadership roles

Whenever there is a crisis, new leadership and new collaborations emerge. For instance, India played a key leadership role during the COVID-19 pandemic. As part of the Vaccine Maitri (Vaccine Friendship) initiative, the Indian Government delivered around 162.9 million doses of vaccines to 96 countries around the world [12–14]. Of these, 14.3 million doses were gifted to various countries and the remaining doses were supplied by the vaccine producers under its commercial and COVAX obligations.

### Harnessing and retaining local talent

There is no dearth of local talent in terms of research and innovation in the Global South which includes the African and the Asian sub-continent. This probably would be a good opportunity to take the South—South Collaboration to a next level, by exploring possibilities of newer models of collaboration, co-funding, co-creation, and co-management of health and development sector projects.

## Leveraging the local innovation and start-up ecosystems

Innovation spending in the Global South countries, although rising year-on-year, varies significantly from country to country. For instance, India’s Research and Development (R&D) spending is around 0.7% of its gross domestic product (GDP), as compared to Israel’s 6.3% of its GDP on R&D and innovation. USA, while having the highest absolute R&D expenditure, spends 3.4% of its GDP on R&D [15]. Countries like India, China, Russia, Brazil, and South Africa probably have the bandwidth to increase funding on health care and innovation.

Leveraging the ever-growing innovation and start-up ecosystem in the med-tech, digital health, wellness, bio-manufacturing, science and technology arena, is another good opportunity that can be accelerated to gain optimum benefits. Several of the Global South countries are already enhancing and expanding their Start-up and Innovation Ecosystems. A rejuvenated focus on the start-up ecosystem, academia and industry collaborations should pay dividends in the future. More impetus to the innovation and start-up ecosystems in the health and allied sectors, to focus on novel, cost-effective and cost-efficient models of programme implementation is essential at this time.

### To increase the per capita GDP on health and development sector issues

The average GDP per capita (in current USD) of some of the Global South Countries varies significantly. The GDP per capita (in current USD) of India is 2696.7 USD, South Africa 6253.4 USD, and China 13 303.1 USD, while for developed countries like USA, it is 85 809.9 USD, for UK it is 52 636.8 USD, for Australia it is 64 407.5 USD, and for Canada it is 54 282.6 USD [16]. On a comparative note, the current health expenditure as a percentage of GDP of some of the developed countries USA, UK, Canada, Germany, and France are 16.50%, 10.87%, 11.22%, 11.80%, and 11.88%, respectively. For countries from the Global South it is 3.31% (India), 5.37% (China), 3.89% (other Lower middle income countries), 5.28% (other middle income countries) [17].

This is an opportune time for countries and governments to reassess their strategies and to start increasing the percentage share of GDP towards Health, Wellness and Development issues. An example here could be the Ayushman Bharat Pradhan Mantri Jan Arogya Yojana (Prime Minister’s people’s Health Scheme), a national public health insurance scheme of the Government of India that aims to provide free access to health insurance coverage for the Below Poverty Line people in the country. This was later expanded to include all citizens above 70 years of age, regardless of their economic status [18–20].

### Miscellaneous immediate measures

### The various country governments should look at the following as a priority.

(i) A rapid assessment of the ongoing USAID supported development sector projects in their respective countries, the absolute and relative need of those projects and also innovative ways and strategies of taking these forward without the pre-existing funding support.
(ii) A conscious effort to be made by the national governments to integrate the ongoing projects previously supported by USAID into the mainstream government programmes. This might also be looked at as an effort to rationalize vertical programmes (run by donor agencies) and integrate them into main stream horizontal programmes.
(iii) An emergency pool of funds could be designed and executed by the respective countries, to support critical areas of health and development—for instance, a fund to support HIV/AIDS investigations and ART, Tuberculosis and Malaria treatment, key maternal, and child health issues and immunization.
(iv) Efforts should be made by local governments and local NGOs to use the local talent (human resource) that existed in these USAID projects—this would be a win–win to both the governments and those that lost their jobs suddenly as a result of the freeze on the USAID projects.
(v) Focusing more on Public Private Partnership Models, wherein the advocacy, policy, and government buy-in could be the responsibility of the Government and the technical know-how and funding could be undertaken by the local Private partner.

While global philanthropy still plays an important role, there is a notable shift from philanthropy to emphasis on Corporate Social Responsibility (CSR) within large conglomerates and businesses. Increasingly businesses are integrating social and environmental consideration into their day-to-day operations and decision-making processes. In many instances the respective Country governments are framing policies in such a manner that funds are allocated and routed through CSR.

It is apparent that Donor Countries, often benefit as much, if not more than the recipient countries, as aid flow contributes to their own economies, gives them access to newer markets (which otherwise they would not have access to), invaluable data and also gives them global political clout. In conclusion, this probably is also an alert to the LMIC country governments that International Aid should not be taken as granted and will definitely not be there for ever. It is better that countries have to look at strategies for self-sustainability, sooner than later. So, a situation like this can be looked at as the right opportunity for exploring locally driven, sustainable, cost-effective, and cost-efficient health and development models.

Strengthening and expansion of geopolitical realignments, newer models of programme implementation, technology enabled monitoring and evaluation that can reduce costs of programme implementation and management would have to emerge. Technical support for these new initiatives can be obtained by the global development sector community more cost-effectively—International agencies like the WHO and United Nations agencies could always be approached for technical guidance and support. This would probably pave the way to developing newer models of implementation science and management.

## Notes

**Srikrishna Sulgodu Ramachandra**, Associate Professor

**Premila Webster**, Professor

## References

[1] Trump orders USA’s exit from WHO, The Guardian. 21 January 2025. https://www.theguardian.com/us-news/2025/jan/20/trump-executive-order-who-withdrawal?CMP=share_btn_url (7 August 2025, date last accessed).

[2] Trump Pulls US From World Health Organization: What You Need to Know. *Newsweek.com*, 22 January 2025. https://www.newsweek.com.world-health-organization-who-trump-pulls-us-2018064 (7 August 2025, date last accessed).

[3] Trump orders US exit from World Health Organization . 22.01.10225, available at https://www.reuters.com/world/us/trump-signs-executive-withdrawing-world-health-organization-2025.01.21/. Last accessed on 2025;07:08.

[4] Adeyi O . Africa without foreign aid for health: free at last? development today, March 2025. https://www.development-today.com/archive/2025/dt-2-2025/africa-without-foreign-aid-for-health-free-at-last (7 August 2025, date last accessed).

[5] Congressional Research Service . Withdrawal from the World Health Organization: Legal Basis and Implications, 2020. https://crsreports.congress.gov/product/pdf/LSB/LSB10489 (7 August 2025, date last accessed).

[6] Kaiser Family Foundation . The U.S. Government and the World Health Organization, 2025. https://www.kff.org/global-health-policy/factsheet/the-u-s-government-and-the-world-health-organization/ (7 August 2025, date last accessed).

[7] Boyce MR, Attal-Juncqua A, Lin J et al. Global Fund contributions to health security in ten countries, 2014-20: mapping synergies between vertical disease programmes and capacities for preventing, detecting and responding to public health emergencies. Lancet Glob Health 2021;9:e181–8. 10.1016/S2214-109X(20)30420-4 33482139 PMC8448292

[8] Yamey G, Titanji BK. Withdrawal of the United States from the WHO – how president trump is weakening public health. N Engl J Med 2025;392(15):1457–60. 10.1056/NEJMp2501790 40043253

[9] Hiscott J, Alexandridi M, Muscolini M et al. The global impact of the coronavirus pandemic. Cytokine Growth Factor Rev 2020;53:1–9. 10.1016/j.cytogfr.2020.05.010 32487439 PMC7254014

[10] Cavalcanti DM, Oliveira Ferreira de Sales L, da Silva AF et al. Evaluating the impact of two decades of USAID interventions and projecting the effects of defunding on mortality up to 2030: a retrospective impact evaluation and forecasting analysis. The Lancet 406:283–94. 10.1016/S0140-6736(25)01186-9 (7 August 2025, date last accessed). PMC12274115 40609560

[11] Rubio says 83% of USAID programs terminated after six-week purge, The Guardian, https://www.theguardian.com/us-news/2025/mar/10/marco-rubio-usaid-funding?CMP=share_btn_url (7 August 2025, date last accessed).

[12] Vaccine Maitri: Consignment of covid vaccines airlifted for Guyana, Jamaica, *mint*, 5 March 2021. https://www.livemint.com/news/india/vaccine-maitri-consignment-of-covid-vaccines-airlifted-for-guyana-jamaica-nicaragua-11614930238803.html (7 August 2025, date last accessed).

[13] Vaccine Maitri: Ministry of External Affairs – Government of India.

[14] Vaccine Maitri: A Sanjeevini for the world. *The Hindu Business Line*. 4 March 2021.

[15] World Bank World Development Indicators . https://data.worldbank.org/indicator/GB.XPD.RSDV.GD.ZS (7 August 2025, date last accessed).

[16] World Health Organization . *Global Spending on Health: Emerging from the Pandemic*. World Health Organization, 2024, https://iris.who.int/handle/10665/379750. License: CC BY-NC-SA 3.0 IGO (7 August 2025, date last accessed).

[17] World Health Organization . *Global Health Expenditure Database*. WHO, www.aps.who.int/nha/database (7 August 2025, date last accessed).

[18] About PM-JAY – National Health Authority/GOI. www.nha.gov.in (7 August 2025, date last accessed).

[19] Ayushman Bharat on way to become world’s largest free healthcare: Arun Jaitley. *The Economic Times. PTI*. 6 March 2019.

[20] About Pradhan Mantri Jan Arogya Yojana (PM-JAY). Ayushmancarddownloadupin. https://nha.gov.in/PM-JAY (7 August 2025, date last accessed).

